# Substantial projections from the lateral division of the lateral habenula to the dorsal raphe nucleus and from the lateral habenula to the contralateral ventral tegmental area

**DOI:** 10.1016/j.heliyon.2024.e40234

**Published:** 2024-11-07

**Authors:** Tomoya Nakamura, Trang Van Thi Nguyen, Takumi Maeda, Hiroyuki Ichijo

**Affiliations:** a Department of Anatomy, Faculty of Medicine, University of Toyama, Japan; b Research Centre for Idling Brain Science, University of Toyama, Japan

**Keywords:** Lateral habenula, Dorsal raphe nucleus, Ventral tegmental area, Retrograde tracer

## Abstract

The lateral habenular nucleus (LHb) projects to the dorsal raphe nucleus (DRN) and ventral tegmental area (VTA). Prior studies have reported that the medial division of the LHb (LHb-m) mainly projects to the DRN, while the LHb mainly projects to the ipsilateral VTA; however, due to only a few studies of projection ratio analysis, the degree of projection of minor and major pathways remains unclear, and the potential significance of minor pathways may be overlooked. After injecting the retrograde tracer into the mice DRN, the proportion of labeled neurons was 63.50 % in the LHb-m and 36.50 % in the lateral division of the LHb (LHb-l). The proportion distributions of labeled neurons were 26.90 % in the anterior LHb-m and 73.10 % in the posterior LHb-m. When the retrograde tracer was injected into the VTA, the percentage of labeled neurons was 64.85 % in the ipsilateral LHb and 35.15 % in the contralateral LHb. The number of cells projecting from anterior and posterior ipsilateral LHb-m to the VTA varied among the individual injection sites. Our quantitative analysis with multiple subjects showed that the projections from LHb-l to DRN and from LHb to contralateral VTA were indeed minor pathways, however, they were substantial. These results provide an anatomical basis for the potential importance of minor pathways projected from the LHb to the VTA and DRN and for the difference in the densities of neurons projecting from the anterior and posterior LHb-m to the DRN.

## Highlights

• The proportion of major and minor pathways were elucidated from the LHb to the DRN and VTA.
• The labeled cells in the LHb were analyzed by using mice injected with a retrograde tracer into the DRN and VTA.
• A substantial number of projects were confirmed from LHb-l to the DRN, and from the LHb to the contralateral VTA.
• The potential significance of the minor pathways from the LHb to the VTA and DRN was suggested.

## 1 Introduction

The lateral habenular nucleus (LHb) is a dorsal diencephalic nucleus that processes negative emotions. Stressful stimuli in early life and adulthood, such as maternal deprivation, physical restraint, and social defeat, have been shown to activate the lateral habenular nucleus (LHb) in rodents [[1], [2], [3], [4]]. In experience-dependent plasticity, LHb maturation is affected by early life stress on postnatal days 10–20 (P10-20) in mice, leading to late effects of hyperactivity in the LHb and anxiety or depression in adulthood [1]. LHb overactivity has been associated with chronic stress-induced anxiety and depression in animal models of anxiety and depression, as well as in patients with depression [[5], [6], [7], [8], [9]]. These findings indicate that LHb overactivity is involved in the development of anxiety and depression. As insufficient secretion of serotonin and dopamine from monoaminergic neurons is involved in anxiety and depression, the neural circuits in which the LHb modulates the dorsal raphe nucleus (DRN) of the serotonergic system and the ventral tegmental area (VTA) of the dopaminergic system have been the focus of significant attention.

The LHb neurons exert inhibitory and some excitatory modulation on the DRN and VTA via direct and indirect neuronal circuits. The projection neurons from the LHb to the DRN are glutamatergic [[10], [11], [12], [13], [14]]. It has been reported that optogenetic stimulation of excitatory LHb-DRN projection neurons increases neuronal activity in the DRN [14]. Conversely, electrical stimulation of the LHb suppresses the firing rate of serotonin neurons in the DRN [[15], [16], [17]], and electrolytic ablation of the LHb increases serotonin levels in the DRN [18]. Furthermore, excitatory neurons in the LHb, whose axon terminals connect dopaminergic and GABAergic neurons, inhibit dopaminergic neurons in the VTA [[19], [20], [21], [22]]. They further project to GABAergic neurons in the rostromedial tegmental nucleus (RMTg), which subsequently project to the VTA [[23], [24], [25]]. Optogenetic activation of the excitatory LHb-VTA projection neurons activated 80 % of non-dopaminergic neurons in the RMTg, as well as a small number of dopaminergic neurons in the VTA [24].

The LHb consists of subnuclei and is thought to be parceled into functional regions [[26], [27], [28]]. The LHb is divided into the medial division (LHb-m) and lateral division (LHb-l). The LHb-m has further been reported to mainly project to the DRN, whereas the LHb mainly projects to the ipsilateral VTA [26,[28], [29], [30]]. However, due to only a few studies of projection ratio analysis of these studies, the degree of projection of minor and major pathways remains unclear, and the potential significance of minor pathways may be overlooked. In this study, we administered a retrograde tracer (cholera toxin subunit B) to the DRN or VTA of mice and counted the number of tracer-labeled neurons the LHb-m and LHb-l. The projections from the LHb-l to the DRN as well as the contralateral projections from the LHb to the VTA were found to be substantial by the quantitative analysis.

## 2 Material and methods

### 2.1 Animal care

Wild-type male C57BL/6 J (B6/J) mice (weight: 18.8–29.0g) were purchased from Japan SLC, Inc. (Hamamatsu, Japan). Mice were housed in a temperature-controlled environment (22–25 °C) under a 12/12-h light/dark cycle (lights were turned on at 5:00 and off at 17:00) in groups of a maximum of five littermates per cage.

### 2.2 Cholera toxin subunit B retrograde tracing procedure

At P54-P105, under pentobarbital anesthesia (50 mg/kg body weight, intraperitoneal injection; Kyoritsu Seiyaku Co., Tokyo, Japan), 2 % lidocaine (AstraZeneca, Cambridge, UK) was applied to the skin, after which the male mice were placed in a stereotaxic frame (Narishige, Tokyo, Japan). A hole was created in the skull just above the target area using a hand drill (VOLVERE i7, NSK-Nakanishi, Kanuma, Japan). From there, the 0.5 μL cholera toxin subunit B (CTB) was delivered using IMS-20 (Narishige, Tokyo, Japan) with a 25 μL syringe (model 702 RN, Hamilton company) filled with mineral oil (Sigma, MO, USA), via a stereotaxically positioned 33G stainless steel cannula (C315I, Plastics one, Anaheim, CA) over 10 min. The cannula was left in place 10 min before the injection to wait for the brain to be pushed back and 15 min after the injection to prevent reverse flow. The stereotaxic coordinates were chosen for each target area based on standard atlas coordinates [31]. Specifically, for the DRN, coordinates along the anteroposterior axis from the bregma (AP) −4.48 mm, along lateral axis from the midline (L) 0 mm, along dorsoventral axis ventral from lambda-bregma line (DV) 2.8 mm were chosen (*n =* 3); for the VTA, AP -3.16 mm, L ± 0.5 mm, DV 4.5 mm were chosen (left side, *n =* 3; right side, *n* = 3). Three days after injection, the mice were perfusion-fixed, as described below. The coordinates of the injection sites were confirmed using histological specimens.

### 2.3 Histological procedures

The mice were transcardially perfused with phosphate-buffered saline (PBS), followed by 3.7 % formaldehyde (Wako, Osaka, Japan) in PBS, under deep anesthesia with pentobarbital (50 mg/kg body weight, i.p.; Kyoritsu Seiyaku Co., Tokyo, Japan). Brains were removed and immersed at 4 °C overnight in the same fixative. After washing in PBS, brains were then embedded in gelatin (comprising 16.7 % gelatin and 16.7 % glycerol in PBS), and postfixed in the same fixative for 4 days at 4 °C. Coronal sections of 70-μm thickness were cut using Vibratome (VT1000S, Leica Microsystems, Wetzlar, Germany). Serial sections were collected extending from the LHb-containing anterior portion (at −1.22 from bregma) to the VTA-containing posterior portion (at −3.88 from bregma), or the DRN-containing posterior portion (at −4.95 from bregma) and stored in PBS containing 0.02 % sodium azide at 4 °C until staining[31].

The sections were stained with 4’,6-diamidino-2-phenylindole in 0.5 % Triton X-100 in PBS (PBST) for 10 min (DAPI; 1:10000; D9542, Sigma-Aldrich, St Louis, USA), and mounted on glass slides with Mowiol 4–88 (Merck Millipore, Burlington, USA). Images were acquired using a confocal laser-scanning microscope (LSM780; Zeiss, Jena, Germany), equipped with a 20 × fluorescent objective lens. A series of confocal Z-stack images were recorded at 2 μm intervals with an X/Y resolution of 708,49 × 708,49 μm (1024 × 1024 pixels).

By observing the cytoarchitecture with DAPI, the medial and lateral divisions of the LHb (LHb-m and LHb-l) were defined following the subnuclear organization of the LHb outlined by Andres et al.[32]. The LHb-m included the anterior, central, marginal, parvocellular, and superior parts, while the LHb-l included the basal, magnocellular, marginal, oval, and parvocellular parts. In all images, the number of CTB-retrogradely labeled neurons was counted using the ImageJ cell counter plug-in (National Institutes of Health, Bethesda, MD, USA; https://www.nih.gov/; https://imagej.nih.gov/ij/plugins/cell-counter.html).

### 2.4 Quantitative analysis of the retrogradely labeled neurons densities in LHb

After the preparation of the histological specimens, labeled neurons were counted in the LHb-m and LHb-l in animals treated with the CTB retrograde tracer in the DRN and VTA. Densities of the labeled neurons (cells/mm^2^) were calculated in three 70-μm sections, with an interval of 210 μm between AP -1.34 and AP -1.97. The coordination of DRN and VTA was identified according to Paxinos and Franklin [31] The injection site and spread of the tracer varied owing to individual differences in the size and shape of mouse skulls or variations in technique. These dispersions can affect the number and distribution of the labeled neurons.

The relative values of the labeled neurons in the LHb-m and LHb-l were calculated from the counted data to correct errors from the injection spread and positional variations between subjects on the number of labeled neurons. In cases of injection of the tracer in the DRN, to observe the mediolateral differences, the relative value of the labeled neurons in the LHb-m was obtained from the labeled cell density in the LHb-m, divided by the labeled cell density across the LHb, which spans from anterior to posterior and left to right for each individual (entire LHb). The relative value of labeled neurons in the LHb-l was obtained from the labeled cell density in the LHb-l, divided by the labeled cell density in the entire LHb. To observe the left and right differences, the following relative values were calculated: left LHb (ratio of the density in the left LHb to the entire LHb) and right LHb (ratio of the density in the right LHb to the entire LHb). In cases where the tracer was injected in the VTA, the following relative values were calculated to observe the mediolateral differences: ipsilateral LHb-m (the ratio of the density in the ipsilateral LHb-m to the ipsilateral LHb), ipsilateral LHb-l (the ratio of the density in the ipsilateral LHb-l to the ipsilateral LHb), contralateral LHb-m (the ratio of the density in the contralateral LHb-m to the contralateral LHb), and contralateral LHb-l (the ratio of the density in the contralateral LHb-l to the contralateral LHb). In addition, the following relative values were calculated to observe the differences between the ipsilateral and contralateral sides: ipsilateral LHb (ratio of density in the ipsilateral LHb to the entire LHb) and contralateral LHb (ratio of density in the contralateral LHb to that in the entire LHb).

### 2.5 Statistical analysis

Data were expressed as the mean ± standard deviation. In Section 3.3, the SD/means were compared between the relative values and densities using a paired *t*-test. Analyses were performed using Microsoft Excel (Microsoft, Washington, USA).

## 3 Results

### 3.1 Major projection from LHb-m and minor significant projection from the LHb-l to the DRN

**Fig. 1:**
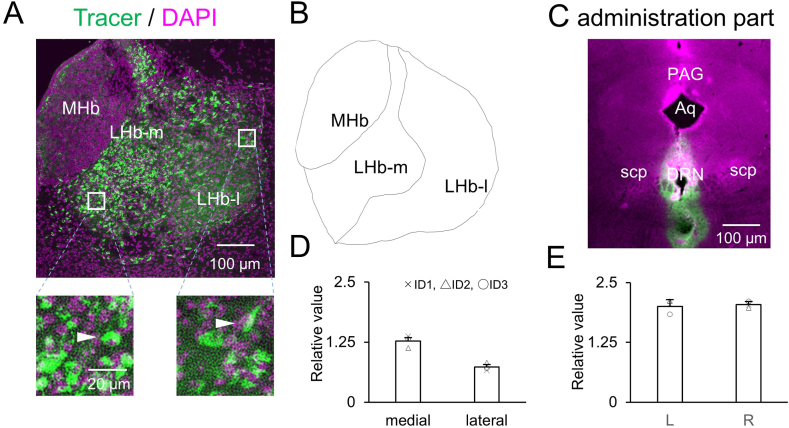
**Projection from the LHb-m and -l to the DRN.** (**A**) Cells labeled with the retrograde tracer CTB (green) and DAPI (magenta) are shown at low magnification in the LHb-m and -l; enlarged images of the boxed areas in LHb-m and -l are further shown. (**B**) Anatomical map of the LHb-m and -l. (**C**) The injection site and spreading of the retrograde tracer in the DRN; the injection was centered in the DRN at AP: −4.84 mm, L: 0 mm, DV: 2.7 mm. (**D**) The relative values of retrograde tracer-labeled neurons in the LHb-m and LHb-l. (**E**) The relative values of retrograde tracer-labeled neurons in the left and right LHb. The cross mark represents the mouse of ID1, the triangle represents ID2 and the white circle represents ID3. Scale bar = 100 μm in low magnification images and 20 μm in high magnification images. Error bars represent the standard error of the mean. LHb; lateral habenula nucleus, MHb; medial habenula nucleus, LHb-m; medial division of the LHb, LHb-l; lateral division of the LHb, DRN; dorsal raphe nucleus, PAG; periaqueductal gray, Aq; Aqueduct, scp; superior cerebellar peduncle. (For interpretation of the references to colour in this figure legend, the reader is referred to the Web version of this article.)

A large number of labeled neurons was observed in the LHb-m and LHb-l when the tracer was injected into the DRN (Fig. 1A and B). The brain coordinates of the injection sites ranged from 0 mm to 0.1 mm in ML, 2.7 mm in DV, and from −4.36 to −4.84 mm in the AP (*n* = 3) (Fig. 1C and Supplementary Fig. 1). The tracer spread area in these slices ranged from 2.16 to 4.68 mm^2^. The labeled cell densities were 2713.462 ± 1423.36 cells/mm^2^ and 1617.00 ± 962.5 cells/mm^2^ in the LHb-m and LHb-l, respectively (mean ± SD, *n =* 3). The relative values of labeled neurons were 1.27 ± 0.13 and 0.73 ± 0.084 in the LHb-m and LHb-l, respectively (mean ± SD, *n* = 3) (Fig. 1D). Thus, the percentage distributions of labeled neurons were 63.50 % (1.27/[1.27 + 0.73]) in the LHb-m and 36.50 % (0.73/[1.27 + 0.73]) in the LHb-l (Table 1). These results indicate that although the percentage of labeled neurons in the LHb-m was large, a substantial percentage of labeled neurons were also observed in the LHb-l.

**Table 1:** **SD/mean values and each experimental data.** LHb; lateral habenula nucleus, LHb-m; medial division of the LHb, LHb-l; lateral division of the LHb, DRN; dorsal raphe nucleus, VTA; ventral tegmental area.

		SD/mean values (%)		Relative values		Densities	
Injected areas	Counted areas	Relative values	Densities	Mean values	SD values	Mean values	SD values
DRN	LHb-m	10.13006404	52.45561321	1.27	0.13	2713.46	1423.36
	LHb-l	11.41363389	59.52961883	0.73	0.08	1617.00	962.59
	left LHb	7.171851676	51.20343365	2.00	0.14	4247.51	2174.87
	right LHb	3.290953496	56.14915743	2.04	0.07	4468.14	2508.82
Left VTA	ipsilateral LHb-m	20.81407433	31.63496262	1.32	0.27	2382.50	753.70
	ipsilateral LHb-l	24.19131753	31.10614896	0.74	0.18	1326.29	412.56
	contralateral LHb-m	7.495647105	44.05203269	1.45	0.11	1337.09	589.02
	contralateral LHb-l	16.73944302	58.04051178	0.66	0.11	645.16	374.45
Right VTA	ipsilateral LHb-m	24.73571274	50.54381945	1.58	0.39	1434.36	724.98
	ipsilateral LHb-l	33.4592671	25.13664515	0.62	0.21	510.83	128.41
	contralateral LHb-m	18.1526129	70.45172664	1.08	0.20	667.37	470.17
	contralateral LHb-l	15.8118766	58.3101948	0.93	0.15	504.55	294.20
	Average	16.11720454	49.05115543				
	SD	8.758028689	13.56933492				

In contrast, the number of labeled neurons in the left and right LHb were similar, with densities of 4247.51 ± 2174.87 cells/mm^2^ and 4468.14 ± 2508.82 cells/mm^2^, respectively (mean ± SD, *n =* 3). The relative values of labeled neurons were 2.00 ± 0.14 and 2.04 ± 0.067 in the left and right LHb, respectively (mean ± SD, *n* = 3) (Fig. 1E).

### 3.2 Major projection from the ipsilateral LHbs and minor significant projection from the contralateral LHbs to the VTA

**Fig. 2:**
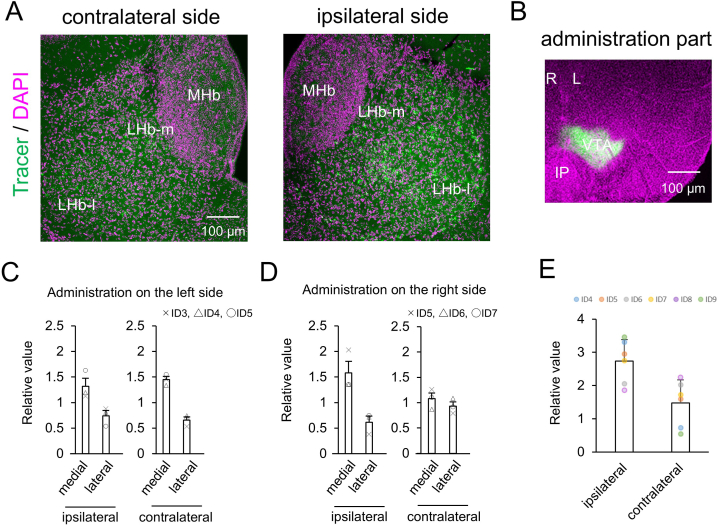
**Bilateral projections from the LHbs to the unilateral VTA.** (**A**) Cells labeled with the retrograde tracer CTB (green) and DAPI (magenta) are shown in the ipsilateral (left) and contralateral (right) LHb. (**B**) The injection site and spreading of retrograde tracer in the left VTA; the injection was centered in the left VTA at AP: −3.66 mm, L: −0.5 mm, DV: 4.3 mm in this case. (**C**) The relative values of tracer-labeled neurons in the medial and lateral division of the ipsilateral and contralateral LHb projecting to the left VTA. The cross mark represents the mouse of ID4, the triangle represents ID5 and the white circle represents ID6. (**D**) The relative values of tracer-labeled neurons in the medial and lateral division of the ipsilateral and contralateral LHbs projecting to the right VTA. The cross mark represents the mouse of ID7, the triangle represents ID8 and the white circle represents ID9. (**E**) The relative values of tracer-labeled neurons in the ipsilateral and contralateral LHbs projecting to the VTA. The blue circle represents the mouse of ID4, the orange circle represents ID5, the gray circle represents ID6, the yellow circle represents ID7, the purple circle represents ID8, and the green circle represents ID9. Scale bar = 100 μm. Error bars represent the standard error of the mean. LHb; lateral habenula nucleus, MHb; medial habenula nucleus, LHb-m; medial division of the LHb, LHb-l; lateral division of the LHb, VTA; ventral tegmental area, IP; interpeduncular nucleus, L = left, R = right. (For interpretation of the references to colour in this figure legend, the reader is referred to the Web version of this article.)

Labeled neurons were identified in the LHb-m and LHb-l on the ipsilateral and contralateral sides when the tracer was injected into the VTA (Fig. 2A). The brain coordinates of the injection sites ranged from −3.15 to −3.80 mm in the AP, from ±0.3–0.5 mm in the ML, and from 3.8 to 4.5 mm in the DV (left and right, *n* = 6) (Fig. 2B and Supplementary Fig. 2). The tracer spread area in these slices ranged from 2.43 to 4.98 mm^2^.

When the tracer was injected into the VTA on the left side, the labeled cell densities were 2382.50 ± 753.70 cells/mm^2^, 1326.29 ± 412.56 cells/mm^2^, 1337.09 ± 589.02 cells/mm^2^, and 645.16 ± 374.45 cells/mm^2^ in the ipsilateral LHb-m, ipsilateral LHb-l, contralateral LHb-m, and contralateral LHb-l, respectively (mean ± SD, *n =* 3). When the tracer was injected into the VTA on the right side, the labeled cell densities were 1434.36 ± 724.98 cells/mm^2^, 510.83 ± 128.41 cells/mm^2^, 667.37 ± 470.17 cells/mm^2^, and 504.54 ± 294.20 cells/mm^2^ in the ipsilateral LHb-m, ipsilateral LHb-l, contralateral LHb-m, and contralateral LHb-l, respectively (*n =* 3). When the tracer was injected into the VTA on the left side, the relative values of labeled neurons were 5.75 ± 1.20, 3.24 ± 0.78, 6.32 ± 0.47, and 2.87 ± 0.48 in the ipsilateral LHb-m, ipsilateral LHb-l, contralateral LHb-m and contralateral LHb-l, respectively (*n =* 3) (Fig. 2C). When the tracer was injected into the VTA on the right side, the relative values of labeled neurons were 6.90 ± 1.71, 2.69 ± 0.90, 4.71 ± 0.86, and 4.07 ± 0.65 in the ipsilateral LHb-m, ipsilateral LHb-l, contralateral LHb-m, and contralateral LHb-l, respectively (*n* = 3) (Fig. 2D). Overall, when a retrograde tracer was injected into the left or right VTA, ipsilateral and contralateral labeled neurons were abundant in the LHb-m; however, a substantial number of labeled neurons were also observed in the LHb-l.

The relative values of labeled neurons were 2.73 ± 0.65 in ipsilateral LHbs and 1.48 ± 0.69 in the contralateral LHbs (mean ± SD, *n* = 6) (Fig. 2E). Thus, the percentage distributions of labeled neurons were 64.85 % (2.73/[2.73 + 1.48]) in the ipsilateral LHb and 35.15 % (1.48/[2.73 + 1.48]) in the contralateral LHb (Table 1). These results indicate that although the number of labeled neurons in the ipsilateral LHbs was large, a substantial number of labeled neurons were also observed in the contralateral LHbs.

### 3.3 Differences in cell density distribution projecting to DRN from anterior and posterior LHb-m

**Fig. 3:**
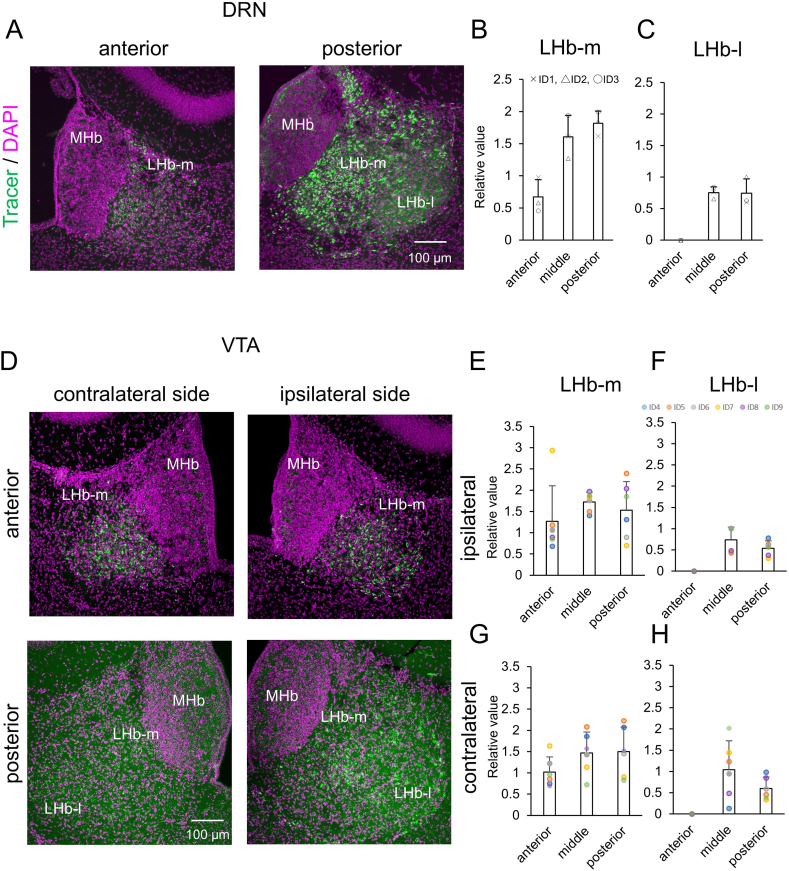
**Anteroposterior distribution of retrograde trace-labeled neurons projecting from LHb to DRN and VTA.** (**A**) Cells labeled with the retrograde tracer CTB (green) injected DRN and DAPI (magenta) are shown at anterior and posterior LHb. The relative values of retrograde tracer-labeled neurons in the anterior, middle, and posterior (**B**) LHb-m and (**C**) LHb-l. The cross mark represents the mouse of ID1, the triangle represents ID2, and the white circle represents ID3. (**D**) Cells labeled with the retrograde tracer CTB (green) injected VTA and DAPI (magenta) are shown at anterior and posterior LHbs. The relative values of retrograde tracer-labeled neurons in the anterior, middle, and posterior (**E**) ipsilateral LHb-m, (**F**) ipsilateral LHb-l, (**G**) contralateral LHb-m, and (**H**) contralateral LHb-l. The blue circle represents the mouse of ID4, the orange circle represents ID5, the gray circle represents ID6, the yellow circle represents ID7, the purple circle represents ID8, and the green circle represents ID9. Scale bar = 100 μm. Error bars represent the standard error of the mean. LHb; lateral habenula nucleus, MHb; medial habenula nucleus, LHb-m; medial division of the LHb, LHb-l; lateral division of the LHb. (For interpretation of the references to colour in this figure legend, the reader is referred to the Web version of this article.)

The numbers of labeled neurons were compared between the anterior (from AP -1.34 to −1.41), middle (from AP -1.62 to −1.69), and posterior (from AP -1.90 to 1.97) LHb when the tracer was injected into the DRN (Fig. 3A). The relative values of labeled neurons were 0.67 ± 0.27, 1.61 ± 0.33, and 1.82 ± 0.19 in the anterior, middle, and posterior LHb-m (mean ± SD, n = 3) (Fig. 3B), and 0.00 ± 0.00, 0.75 ± 0.09, and 0.74 ± 0.23 in the anterior, middle, and posterior LHb-l (mean ± SD, n = 3 and Supplementary Table) (Fig. 3C). Following the definition of Andres et al. [32] all positive cells in the anterior LHb were counted as belonging to LHb-m. Thus, the percentage distributions of labeled neurons were 26.90 % (0.67/[0.67 + 1.82]) in the anterior LHb-m and 73.10 % (1.82/[0.67 + 1.82]) in the posterior LHb-m. These results indicate that the posterior LHb-m has more neurons projecting to the DRN than the anterior.

The numbers of labeled neurons were compared between the anterior (from AP -1.34 to −1.41), middle (from AP -1.62 to −1.69), and posterior (from AP -1.90 to 1.97) LHb when the tracer was injected into the VTA (Fig. 3D). The relative values of labeled neuron were 1.27 ± 0.84, 1.73 ± 0.23, and 1.53 ± 0.67 in the anterior, middle, and posterior ipsilateral LHb-m (mean ± SD, n = 6) (Figs. 3E), 0.00 ± 0.00, 0.74 ± 0.30, and 0.54 ± 0.17 in the anterior, middle, and posterior ipsilateral LHb-l (mean ± SD, n = 6) (Figs. 3F), 1.02 ± 0.35, 1.47 ± 0.49, and 1.50 ± 0.58 in the anterior, middle, and posterior contralateral LHb-m (mean ± SD, n = 6) (Fig. 3G), and 0.00 ± 0.00, 1.04 ± 0.68, and 0.60 ± 0.26 in the anterior, middle, and posterior contralateral LHb-l (mean ± SD, n = 6) (Fig. 3H and Supplementary Table). The relative number of cells projecting from anterior and posterior ipsilateral LHb-m to the VTA has a large SD value, and this variability possibly results from individual differences in the injection site.

### 3.4 Lower variability of the relative value

The availability of the relative values used in this study was assessed. The variabilities in the relative values and densities of labeled cells were compared by calculating the ratio of the SD to the mean (Table 1). When the tracer was injected into the DRN, the SD/mean of relative values and densities were 10.13 % and 52.46 % in the LHb-m, 11.41 % and 59.53 % in the LHb-l, 7.18 % and 51.12 % in the left LHb, and 3.30 % and 56.15 % in the right LHb, respectively. When the tracer was injected into the left VTA, the SD/mean relative values and densities were 20.81 % and 31.64 % in the ipsilateral LHb-m, 24.19 % and 31.10 % in the ipsilateral LHb-l, 7.50 % and 44.05 % in the contralateral LHb-m, and 16.73 % and 58.04 % in the contralateral LHb-l. When the tracer was injected into the right VTA, the SD/mean of the relative values and densities were 24.74 % and 50.54 % in the ipsilateral LHb-m, 33.45 % and 25.14 % in the ipsilateral LHb-l, 18.15 % and 70.45 % in the contralateral LHb-m, and 15.81 % and 58.31 % in the contralateral LHb-l. The SD/mean of the relative value (16.12 ± 8.76 %) was significantly smaller than the SD/mean of the density (49.05 ± 13.57 %) (paired *t*-test, *n =* 12, *p* = 0.00013). Overall, these results indicate that the relative values could offset the variations owing to the injection spread and positional variations between multiple subjects.

## 4 Discussion

In the present study, a retrograde tracer was injected into either the DRN or VTA in mice, after which the number of tracer-labeled neurons in the LHb was counted. To examine the proportion of LHb-m and LHb-l cells projecting to the DRN and VTA, the proportion of LHb cells projecting to the ipsilateral or contralateral VTA, and the anteroposterior proportions of LHb cells projecting to the DRN and VTA, quantitative analysis was performed using the relative values to offset variations caused by injection spread or positional variations between multiple subjects.

In this study, a wide range of projection areas were evaluated. This is likely to contribute to understanding the extensive projection patterns of the DRN and VTA neural circuits. However, the influence of tracers outside the target area would be difficult to completely eliminate. When the tracer was administered to the DRN, it possibly spread to the superior cerebellar peduncle and median raphe nucleus, etc., which projected from LHb [33], in ID1 and ID2 mice (Supplementary Figs. 1A and 1B). Although the tracer spread was observed only within the DRN in ID3 (Supplementary Fig. 1C), the distribution patterns of tracer-labeled neurons in the mice of ID1-3 were similar in each individual (Supplementary Table). Therefore, even if the tracer administered to the DRN spreads to other regions, its impact is considered small. When the tracer was administered to the VTA, it possibly spread to the contralateral side of the VTA, although sufficiently less ipsilateral VTA, and ipsilateral to the interpeduncular nucleus (IPN) and the region above VTA (Supplementary Fig. 2). In the previous paper that administers an anterograde virus to the LHb and comprehensively examines projection sites, projections to the IPN in the vicinity of the VTA are observed, fewer neurons project from the LHb to the IPN than to the VTA, and no projections from the LHb are observed in the area above the VTA [33]. Therefore, even if the tracer administered to the VTA had spread to other regions, its impact is considered small.

This study showed that the DRN received projections mainly (63.3 %) from the LHb-m, and the rest (36.7 %) from the LHb-l, as demonstrated by retrograde tracer administration to the DRNs (Fig. 1D). Previous studies using retrograde tracers in the DRN have further shown that the DRN receives projections mainly from the LHb-m and partly from the LHb-l in mice and rats [[26], [27], [28]] which is generally consistent with our results. However, the projection from the LHb-l to the DRN has not been depicted in schematic diagrams in previous reports [26,27,34,35]. Moreover, the present results indicate that the projection from the LHb-l to the DRN is sufficiently large to raise attention, as 36.7 % is not negligible.

In the present study, when retrograde tracers were comprehensively administered to both the dorsal and ventral sides of the VTA, LHb-m cells were confirmed to project predominantly to the ipsilateral VTA and LHb-l cells to both sides of the VTA (Fig. 2B, C, D, and E). A previous study indicated that the LHb-l projects to the anteroventral and posterior parts of the ipsilateral VTA, while the LHb-m projects to the anterodorsal, ventral, and posterior parts of the ipsilateral VTA in rats [36]. In mice, the LHb-l mainly projects to the ipsilateral VTA and RMTg, while partial projections from the LHb-l to the contralateral VTA and RMTg and from the LHb-m to the ipsilateral and contralateral VTA and RMTg have also been shown [28]. Although the boundary between the VTA and RMTg remains poorly clarified, the caudal pole of the VTA is known to contain a region called the RMTg. Previous studies in which retrograde tracers were separately administered to the centers of the VTA and RMTg in rats reported projections from the ipsilateral LHb-m, mainly to the VTA, and from the ipsilateral LHb-l, mainly to the RMTg [27,29,36]. As shown above, LHb projects bilaterally to the VTA and RMTg, although the ratio of ipsilateral to contralateral depends on whether the retrograde tracer was administered ventrally, dorsally, or anteroposteriorly, as well as on the animal species. Further research is required to elucidate these intricate differences. The results obtained from multiple samples present study were consistent with those of previous studies, considering that the retrograde tracer was administered to a broad region of the VTA.

This study showed that both the LHb-m and LHb-l projected onto the VTA on both sides, similar to previous studies [26,28,33,36] (Fig. 2E). Furthermore, 64.8 % and 35.2 % of labeled neurons were located in the ipsilateral and contralateral LHb, respectively (Fig. 2E). These results were confirmed in multiple subjects (*n* = 6), reinforcing the findings of previous reports describing projection proportions performed on only a single subject [28]. It has further been suggested that the projection from the LHb to the contralateral VTA is sufficiently large to warrant attention, as 35.2 % is not negligible.

This study indicated the posterior LHb-m had more neurons projecting to the DRN than the anterior. (Fig. 3). The number of cells projecting from anterior and posterior ipsilateral LHb-m to the VTA was possibly varied among the individual injection site (Fig. 3 and Supplementary Table). These results possibly relate to the subnuclear topographic organization of the LHb [11,32,37] and the subset topography of the cell type of parvalbumin-positive neurons differ in the anteroposterior LHb [38], as well as functional topography after stress stimulation in the LHb [2].

**Fig. 4:**
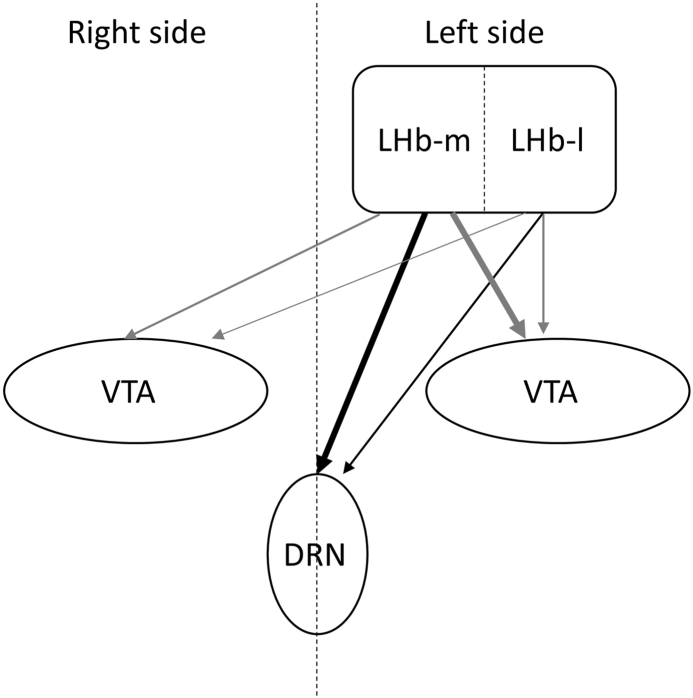
**Schematic representation of projections from the LHb to the DRN and VTA.** The DRN received projections from the LHb-m and LHb-l (e.g. from left LHb). The VTA received projections from the ipsilateral and contralateral LHbs. Bold lines indicate major projection and thin lines indicate minor projections. LHb; lateral habenula nucleus, MHb; medial habenula nucleus, LHb-m; medial division of the LHb, LHb-l; lateral division of the LHb, DRN; dorsal raphe nucleus, VTA; ventral tegmental area.

In this study, the data of multiple subjects were combined and quantified using the relative values, while detailed proportion projections from the LHb to the DRN and VTA are presented. The relative values used in this study were considered useful for obtaining the projection pathway when the tracer was administered over a wide area, because they could offset variations due to injection spread and positional variations between multiple subjects. Due to only a few studies of projection ratio analysis of these studies, the projection pathways from LHb-l to DRN and from LHb to the contralateral VTA have not received much attention as minor pathways. However, quantitative comparisons with the major pathways indicate that these minor pathways are substantial and should be focused on. We emphasize the possible importance of these circuits, as shown in Fig. 4, in promoting an accurate understanding of LHb neural circuits. These results provide an anatomical basis for the potential significance of minor pathways projected from the LHb to the VTA and DRN.

The limitation of this study includes the lack of subdivision at the site of tracer administration prevents identifying changes in data by the site of administration. The study also omits investigating projection routes other than from the LHb to the DRN and VTA, and lacks functional analysis. Our previous study indicates that LHb neurons in adult mice consist of *vglut2*-positive glutamatergic neurons and *gad2*-or *vgat*-positive GABAergic neurons, with no serotonergic, dopaminergic, or cholinergic markers observed [38]. Therefore, LHb-projecting neurons are likely glutamatergic or GABAergic, but the specific type of each neuron remains unknown, posing an important research topic for understanding the LHb projection circuit.

## CRediT authorship contribution statement

**Tomoya Nakamura:** Writing – review & editing, Writing – original draft, Visualization, Validation, Project administration, Methodology, Investigation, Funding acquisition, Formal analysis, Data curation, Conceptualization. **Trang Van Thi Nguyen:** Writing – review & editing, Visualization, Validation, Data curation. **Takumi Maeda:** Writing – review & editing, Validation, Data curation. **Hiroyuki Ichijo:** Writing – review & editing, Validation, Supervision, Project administration, Funding acquisition, Conceptualization.

## Ethics statement

The subjects were treated in strict accordance with the Guidelines for the Care and Use of Laboratory Animals, as approved by the University of Toyama, the US National Institutes of Health Guide for the Care and Use of Laboratory Animals, the Ethics Committee for Animal Experiments at the University of Toyama (license numbers A2017MED-2 and A2019MED-34), and the Animal Research: Reporting of In Vivo Experiments (ARRIVE) guidelines.

## Funding sources

This research was supported by Kakenhi [grant numbers 20K16486 and 21K06371 (10.13039/501100001691JSPS)], a grant-in-aid from Hokuriku Bank, Ltd., and Tamura Science and Technology, Foundation and Research Projects from the 10.13039/100016213University of Toyama.

## Declaration of competing interest

The authors declare that they have no known competing financial interests or personal relationships that could have appeared to influence the work reported in this paper.
