# Supplementary material for: Substantial projections from the lateral division of the lateral habenula to the dorsal raphe nucleus and from the lateral habenula to the contralateral ventral tegmental area
Source: Heliyon. 2024 Nov 7;10(22):e40234. doi: 10.1016/j.heliyon.2024.e40234 (PMC11600024; doi:10.1016/j.heliyon.2024.e40234)
Supplement: Multimedia component 1 [file mmc1.pdf]

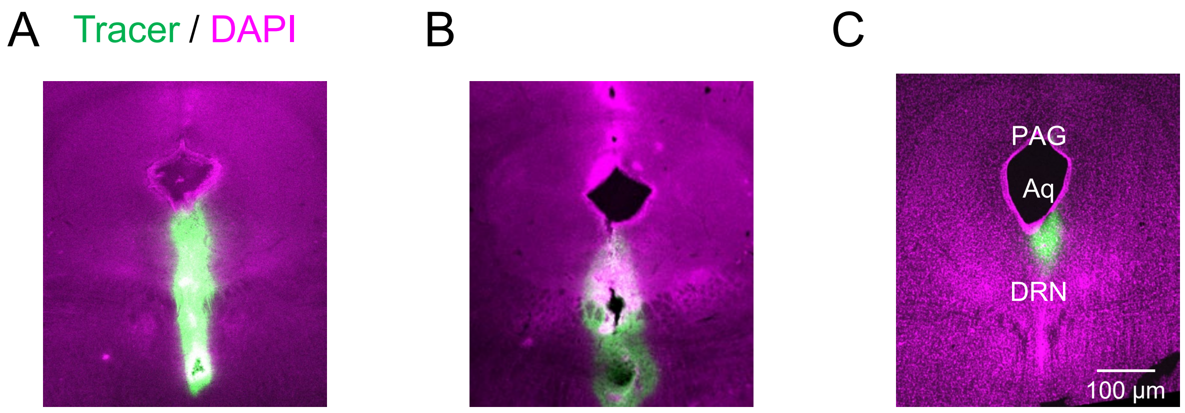


**Supplementary Figure 1.** The injection site and spreading of the retrograde tracer in the DRN. The injection was centered in the DRN at (**A**) AP: -4.59 mm, L: -0.1 mm, DV: 3.2 mm (ID1), (**B**) AP: -4.84 mm, L: 0 mm, DV: 2.7 mm (ID2), (**C**) AP: -4.36 mm, L: -0.1 mm, DV: 2.5 mm (ID3). DRN; dorsal raphe nucleus, PAG; periaqueductal gray, Aq; Aqueduct. Scale bar = 100 µm.

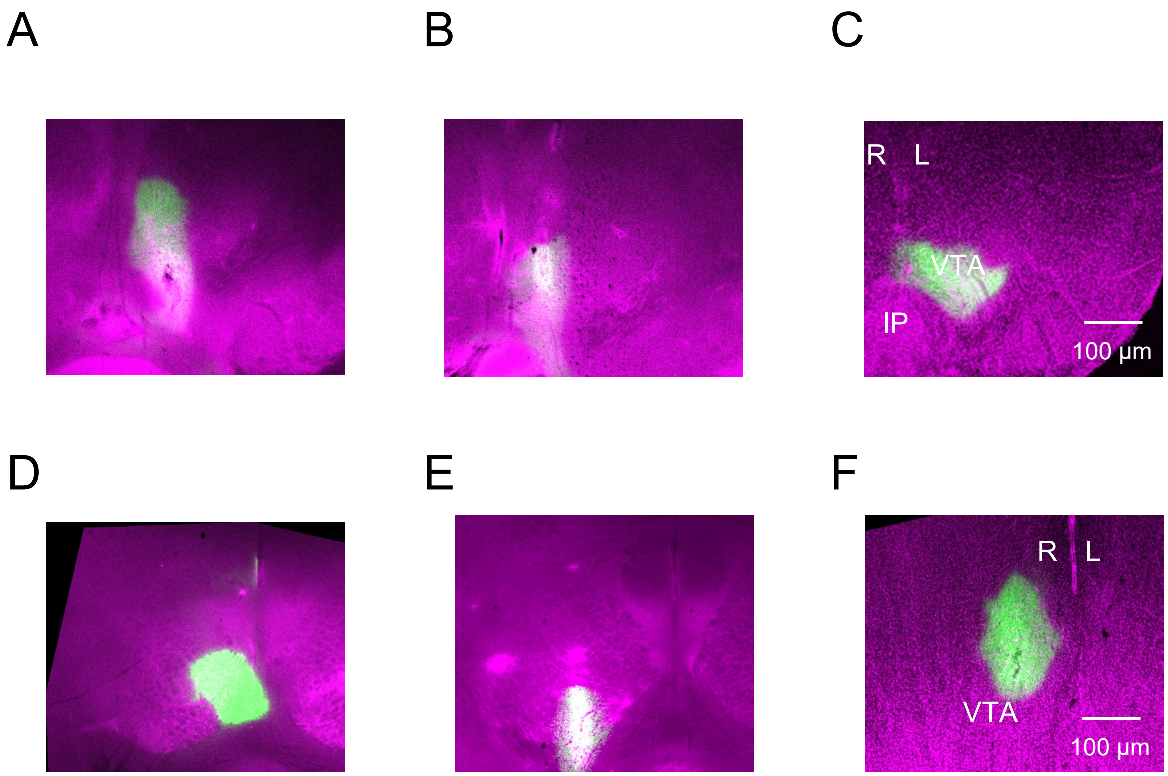


**Supplementary Figure 2. The injection site and spreading of retrograde tracer in the left VTA.** The injection was centered in the left VTA at (A) AP: -3.15 mm, L: -0.5 mm, DV: 4.5 mm (ID4),(B) AP: -3.20 mm, L: -0.3 mm, DV: 4.5 mm (ID5), (C) AP: -3.66 mm, L: -0.5 mm, DV: 4.3 mm (ID6) (D) AP: -3.16 mm, L: 0.4 mm, DV: 4.5 mm (ID7), (E) AP: -3.80 mm, L: -0.5 mm, DV: 4.5 mm (ID8), (F) AP: -3.16 mm, L: -0.4 mm, DV: 3.8 mm (ID9). Scale bar = 100 µm. VTA; ventral tegmental area, IP; interpeduncular nucleus, L = left, R = right.
